# Supplementary material for: Giant anisotropic photonics in the 1D van der Waals semiconductor fibrous red phosphorus
Source: Nat Commun. 2021 Aug 10;12:4822. doi: 10.1038/s41467-021-25104-6 (PMC8355160; doi:10.1038/s41467-021-25104-6)
Supplement: Supplementary file 1 — Supplementary Information [file 41467_2021_25104_MOESM1_ESM.pdf]

# Giant anisotropic photonics in the 1D van der Waals semiconductor fibrous red phosphorus

Luojun Du[1,10]*, Yanchong Zhao[2,3,10], Linlu Wu[4,10], Xuerong Hu[1,5,10], Lide Yao[6], Yadong Wang[1], Xueyin Bai[1], Yunyun Dai[1], Jingsi Qiao[7], Uddin Md Gius[1], Xiaomei Li[2,3], Jouko Lahtinen[6], Xuedong Bai[2,3,8], Guangyu Zhang[2,3,8], Wei Ji[4]*, Zhipei Sun[1,9]*

[1]Department of Electronics and Nanoengineering, Aalto University, Tietotie 3, FI-02150, Finland
[2]Beijing National Laboratory for Condensed Matter Physics; Key Laboratory for Nanoscale Physics and Devices, Institute of Physics, Chinese Academy of Sciences, Beijing 100190, China
[3]School of Physical Sciences, University of Chinese Academy of Sciences, Beijing, 100190, China
[4]Beijing Key Laboratory of Optoelectronic Functional Materials & Micro-Nano Devices, Department of Physics, Renmin University of China, Beijing 100872, P.R. China
[5]Institute of Photonics and Photon Technology, Northwest University, Xi'an 710069, China
[6]Department of Applied Physics, Aalto University, P. O. Box 15100, FI-00076 Aalto, Finland
[7]Centre for Advanced 2D Materials and Graphene Research Centre, National University of Singapore, Singapore 117546, Singapore
[8]Songshan Lake Materials Laboratory, Dongguan, Guangdong, 523808, China
[9]QTF Centre of Excellence, Department of Applied Physics, Aalto University, FI-00076 Aalto, Finland
[10]These authors contributed equally: Luojun Du, Yanchong Zhao, LinLu Wu and Xuerong Hu
*Corresponding author. Email: luojun.du@aalto.fi; wji@ruc.edu.cn; zhipei.sun@aalto.fi

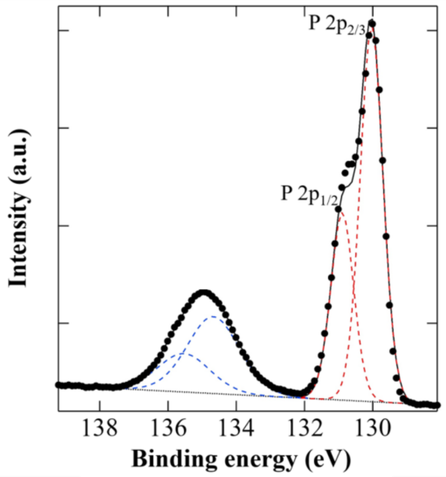


Supplementary Figure 1. High-resolution XPS spectra of the 2*p* orbital of elemental phosphorus.

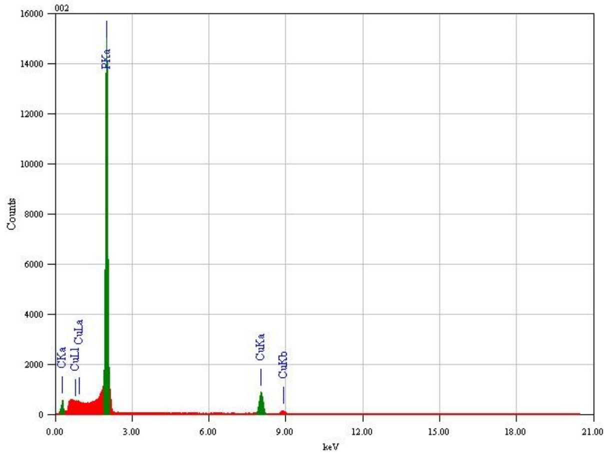


Supplementary Figure 2. Energy dispersive analytical X-ray spectroscopy (EDS) spectrum of FRP.

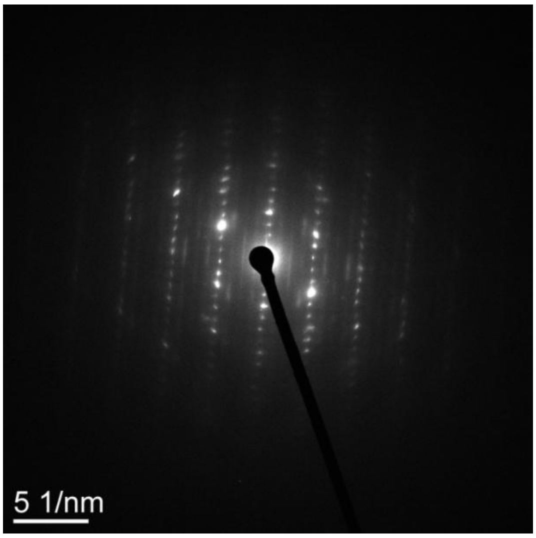


Supplementary Figure 3. Selected-area electron diffraction (SAED) pattern of FRP.

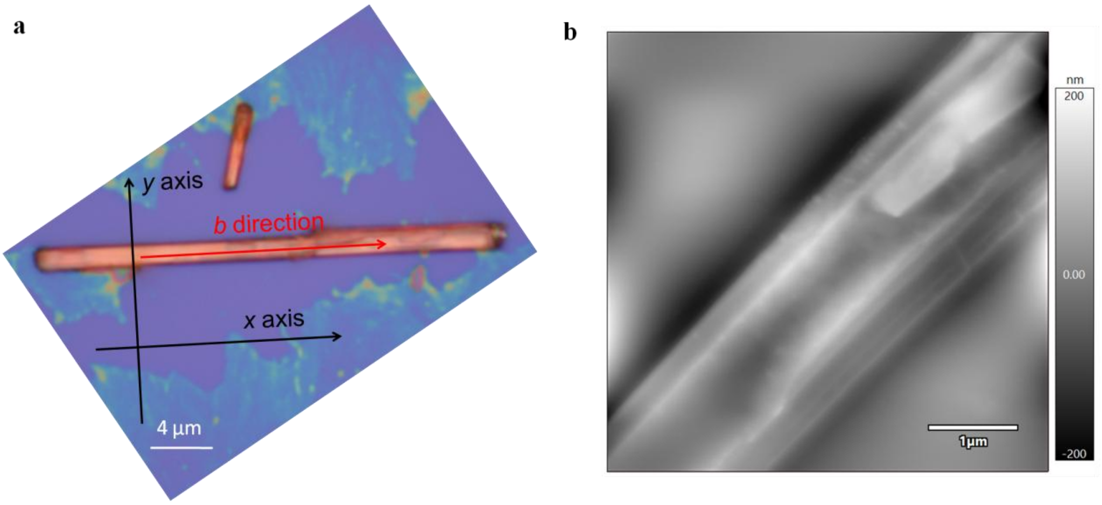


Supplementary Figure 4. (a) Optical microscopy image of FRP. (b) AFM image of FRP with a larger view than Figure 2a in the main text.

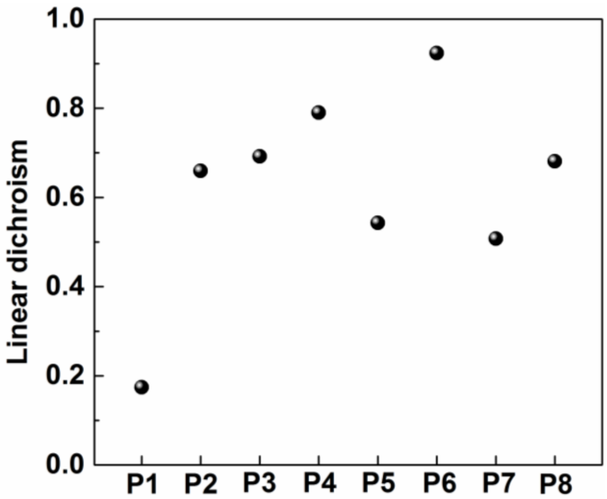


Supplementary Figure 5. The linear dichroism of exciton P1-P8 for polarization angle $\theta = 0°$.

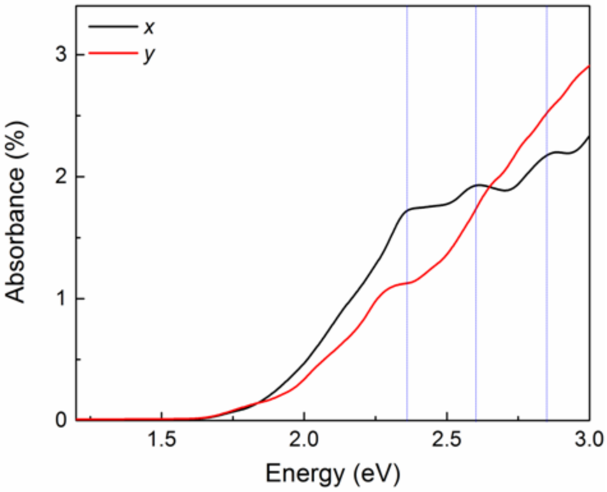


Supplementary Figure 6. Calculated absorption spectra of 1D vdW FRP. Three exciton transitions

with 2.37 eV, 2.61 eV and 2.89 eV are marked by the dashed vertical lines. Since the optB88-vdW DFT functional usually underestimates the bandgap of a semiconductor, the theoretical absorption peaks obtained using optB88-vdW should have lower energies than the experimental peaks, if those peaks originate from the interband transitions without or with very weak many-body electron-hole interactions. On the other hand, if the absorption peaks are from excitons, the calculated absorption spectra with the optB88-vdW functional are, most likely, comparable to experimental results since the excitonic effects can fortuitously cancel the underestimated bandgap that optB88-vdW functional usually shows[1]. Our theoretical absorption spectra show three peaks located at ~ 2.37 eV, 2.61 eV and 2.89 eV, respectively, which are well consistent with the corresponding experimental values of ~ 2.43 eV, 2.55 eV and 2.86 eV [Figure 4b in the main text]. This comparison indicates that these three absorption peaks of FRP may come from excitons. The absorption spectra are calculated from the dielectric function using expression $A(\omega) = \alpha(\omega) \cdot \Delta z$, where $\alpha(\omega) = \frac{\omega Im\varepsilon}{cn}$ is the absorption coefficient, $n = \sqrt{\frac{\sqrt{(Re\varepsilon)^2+(Im\varepsilon)^2}+Re\varepsilon}{2}}$ is the index of refraction, $Re\varepsilon$ and $Im\varepsilon$ are the real and imaginary parts of the dielectric function, respectively[2]. Here, $\omega$ is the light frequency, $c$ is the speed of light in vacuum and $\Delta z$ represents projection of the unit-cell size in the direction perpendicular to $ab$ plane. The conductivity tensor is calculated from the imaginary parts of the dielectric function as well, $\sigma_{ij} = \frac{\omega}{4\pi} Im\varepsilon_{ij}$, where $i$, $j$ represent directions $x$, $y$ or $z$. The electronic structures are obtained from the results unveiled using the optB88-vdW functional and the $k$-mesh is increased to 7×11×11 in calculating dielectric functions. 320 bands are considered in the optical property calculations. Because the dielectric function is a tensor, the absorption spectra along the $x$, $y$ and $z$ directions are obtained separately.

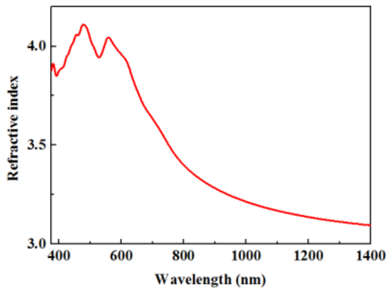


Supplementary Figure 7. Wavelength dependent refractive index of FRP obtained by DFT calculations. For the pump wavelength of 1300 nm, $n_{\omega}$ and $n_{3\omega}$ are 3.11 and 3.98, respectively.

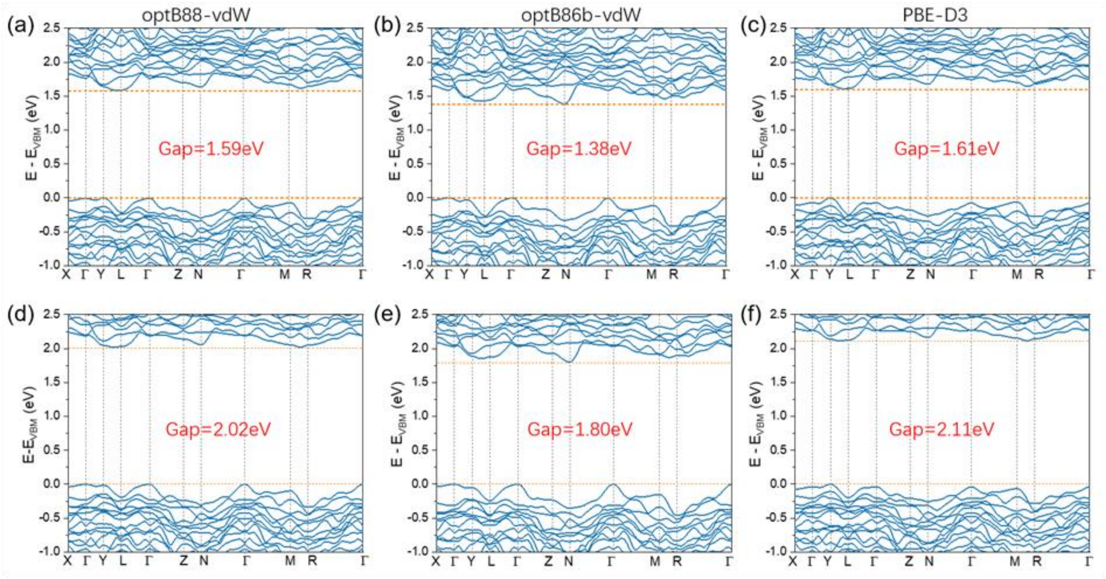


Supplementary Figure 8. Theoretical band structures of FRP with the optB88-vdW (a), optB86b-vdW (b), PBE-D3 (c) and modified Becke-Johnson (mBJ, d-f) functionals. Structures are optimized with the optB88-vdW (a, d), optB86b-vdW (b, e) and PBE-D3 (c, f) functionals, respectively. All these band structures show that FRP is an indirect semiconductor. The mBJ functional shall predict a reliable fundamental bandgap because the errors from over-estimated delocalization of electrons and/or holes are corrected.

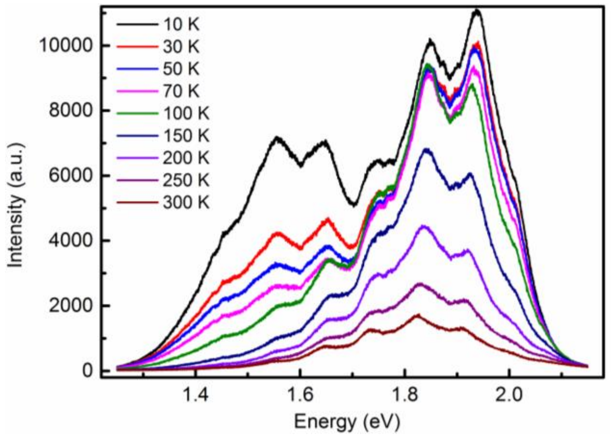


Supplementary Figure 9. Temperature dependent PL spectra of FRP.

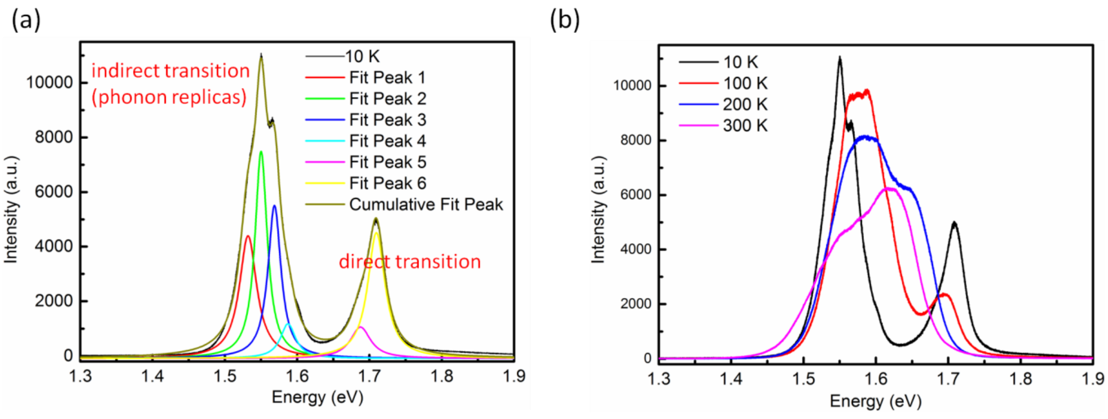

Supplementary Figure 10. (a) PL spectrum of bilayer $WSe_2$ and its fitting at 10 K. (b) Temperature dependent PL spectra of bilayer $WSe_2$. The intensities of phonon replicas increase with decreasing the temperature.

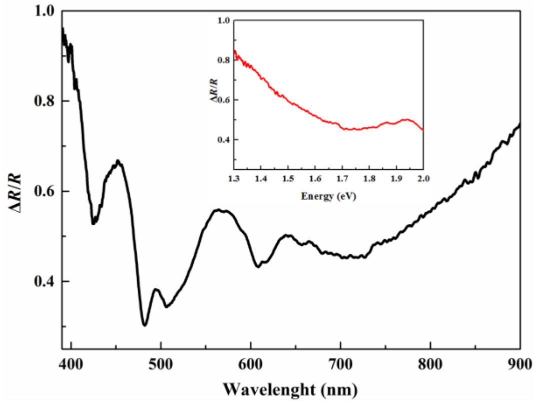


Supplementary Figure 11. Reflectance contrast spectrum in the range of 380-900 nm. In the range of 1.3-2 eV (inset), the reflectance contrast spectrum is relatively smooth and no obvious absorption peak is observed.

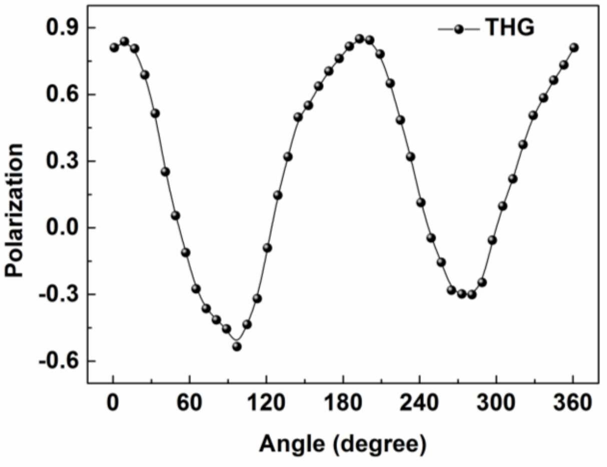


Supplementary Figure 12. Polarization of THG signal as a function of the polarization angle $\theta$.

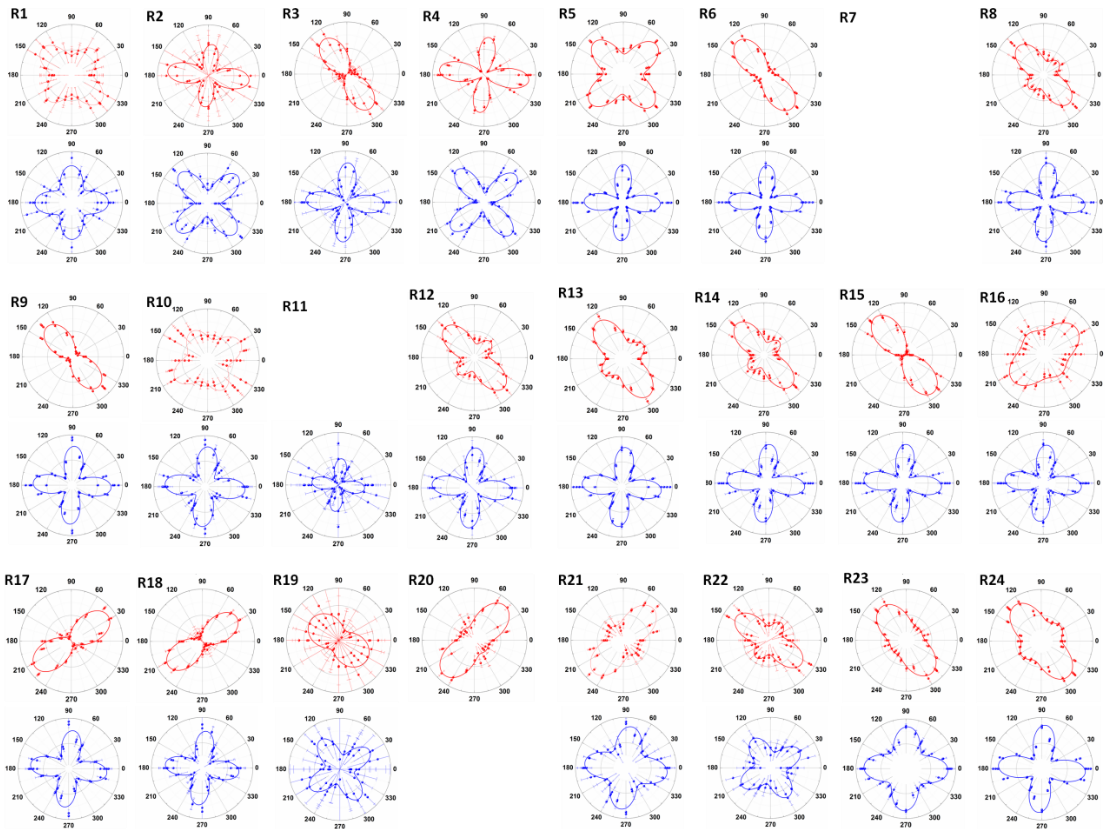


Supplementary Figure 13. The polar plots of Raman intensity for six representative phonons as a function of polarization angle $\theta$ under co-polarized (red) and cross-polarized (blue) configurations.

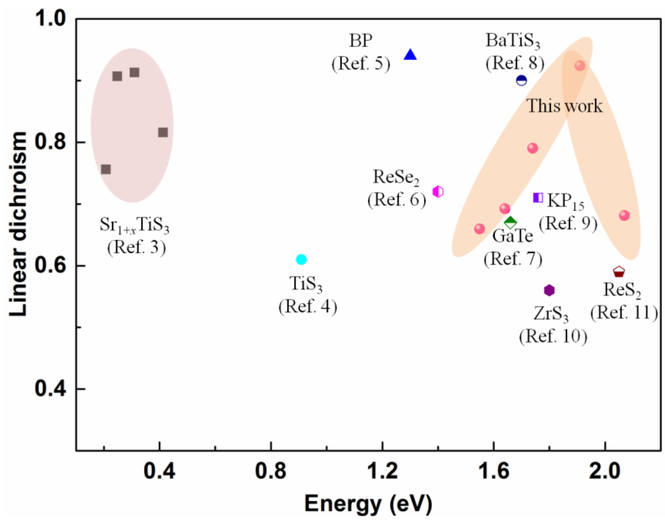


Supplementary Figure 14. The comparison of linear dichroism of FRP with various other typical materials[3-11]. The horizontal axis represents the energy to which the linear dichroism of different materials corresponds. Note that linear dichroism is defined as: linear dichroism $= \frac{I_x - I_y}{I_x + I_y}$, where $I_x$ and $I_y$ denote the photoluminescence emission/absorption/transmission detected in two crystal directions orthogonal to each other. It is clear that linear dichroism should be within the range from $-1$ to 1.

| Supplementary Table 1. The comparison between the THG third-order susceptibility $\chi^{(3)}$ of FRP with various other typical materials. | | |
|---|---|---|
| Material | $\chi^{(3)}$ ($10^{-19}$ $m^2/V^2$) | References |
| FRP | 26.72 | This work |
| $MoS_2$ | 8.99 | This work |
| Graphene | 5-10 | Ref. 12 |
| Graphene-cavity | 400 | Ref. 13 |
| Black phosphorus | 1.6 | Ref. 14 |
| $WS_2$ | 2.4 | Ref. 15 |
| $MoSe_2$ | 2.2 | Ref. 15 |
| GaSe | 1600 | Ref. 16 |
| GaTe | 2000 | Ref. 17 |
| GaAs | 14 | Ref. 18 |
| Gold | 7.6 | Ref. 18 |
| $TiO_2$ | 0.21 | Ref. 18 |
| $LiNbO_3$ | 0.032 | Ref. 19 |

## Supplementary References


1 Rangel, T. *et al.* Large bulk photovoltaic effect and spontaneous polarization of single-layer monochalcogenides. *Phys. Rev. Lett.* **119**, 067402 (2017).

2 Gajdoš, M., Hummer, K., Kresse, G., Furthmüller, J. & Bechstedt, F. Linear optical properties in the projector-augmented wave methodology. *Phys. Rev. B* **73**, 045112 (2006).

3 Niu, S. *et al.* Mid-wave and long-wave infrared linear dichroism in a hexagonal perovskite chalcogenide. *Chem. Mater.* **30**, 4897-4901 (2018).

4 Khatibi, A. *et al.* Anisotropic infrared light emission from quasi-1D layered $TiS_3$. *2D Mater.* **7**, 015022 (2019).

5 Wang, X. *et al.* Highly anisotropic and robust excitons in monolayer black phosphorus. *Nat. Nanotechnol.* **10**, 517 (2015).

6 Arora, A. *et al.* Highly Anisotropic in-Plane Excitons in Atomically Thin and Bulklike 1T′-$ReSe_2$. *Nano Lett.* **17**, 3202-3207 (2017).

7 Cai, H. *et al.* Synthesis of highly anisotropic semiconducting GaTe nanomaterials and emerging properties enabled by epitaxy. *Adv. Mater.* **29**, 1605551 (2017).

8 Wu, J. *et al.* Linear Dichroism Conversion in Quasi-1D Perovskite Chalcogenide. *Adv. Mater.* **31**, 1902118 (2019).

9 Tian, N. *et al.* High anisotropy in tubular layered exfoliated KP15. *ACS Nano* **12**, 1712-1719 (2018).

10 Pant, A. *et al.* Strong dichroic emission in the pseudo one dimensional material $ZrS_3$. *Nanoscale* **8**, 16259-16265 (2016).

11 Wu, S. *et al.* Phase-engineering-induced generation and control of highly anisotropic and robust excitons in few-layer $ReS_2$. *J. Phys. Chem. Lett.* **8**, 2719-2724 (2017).

12 Jiang, T. *et al.* Gate-tunable third-order nonlinear optical response of massless Dirac fermions in graphene. *Nat. Photon.* **12**, 430-436 (2018).

13 Beckerleg, C. *et al.* Cavity enhanced third harmonic generation in graphene. *Appl. Phys. Lett.* **112**, 011102 (2018).

14 Autere, A. *et al.* Rapid and large-area characterization of exfoliated black phosphorus using third-harmonic generation microscopy. *J. Phys. Chem. Lett.* **8**, 1343-1350 (2017).

15 Autere, A. *et al.* Optical harmonic generation in monolayer group-VI transition metal dichalcogenides. *Phys. Rev. B* **98**, 115426 (2018).

16 Karvonen, L. *et al.* Investigation of second-and third-harmonic generation in few-layer gallium selenide by multiphoton microscopy. *Sci. Rep.* **5**, 1-8 (2015).

17 Susoma, J. *et al.* Second and third harmonic generation in few-layer gallium telluride characterized by multiphoton microscopy. *Appl. Phys. Lett.* **108**, 073103 (2016).

18 Boyd, R. W. *Nonlinear optics*. (Academic press, 2019).

19 Autere, A. *et al.* Nonlinear optics with 2D layered materials. *Adv. Mater.* **30**, 1705963 (2018).
